# Antioxidant and Neuroprotective Effects of Caffeine against Alzheimer’s and Parkinson’s Disease: Insight into the Role of Nrf-2 and A2AR Signaling

**DOI:** 10.3390/antiox9090902

**Published:** 2020-09-22

**Authors:** Muhammad Ikram, Tae Ju Park, Tahir Ali, Myeong Ok Kim

**Affiliations:** 1 Division of Life Science and Applied Life Science (BK21 plus), College of Natural Sciences, Gyeongsang National University, Jinju 52828, Korea; qazafi417@gnu.ac.kr (M.I.); tahirneuro@gmail.com (T.A.); 2 Paul O’Gorman Leukaemia Research Centre, Institute of Cancer Sciences, University of Glasgow, Glasgow 0747 657 5394, UK; 2358860P@student.gla.ac.uk

**Keywords:** caffeine, Alzheimer’s disease, Parkinson’s disease, neurodegeneration, neuroprotective effects, antioxidant effects

## Abstract

This paper reviews the results of studies conducted on the role of caffeine in the management of different neurological disorders, such as Parkinson’s disease (PD) and Alzheimer’s disease (AD). To highlight the potential role of caffeine in managing different neurodegenerative diseases, we identified studies by searching PubMed, Web of Science, and Google Scholar by scrutinizing the lists of pertinent publications. According to the collected overall findings, caffeine may reduce the elevated oxidative stress; inhibit the activation of adenosine A2A, thereby regulating the accumulation of Aβ; reduce the hyperphosphorylation of tau; and reduce the accumulation of misfolded proteins, such as α-synuclein, in Alzheimer’s and Parkinson’s diseases. The studies have suggested that caffeine has promising protective effects against different neurodegenerative diseases and that these effects may be used to tackle the neurological diseases and/or their consequences. Here, we review the ongoing research on the role of caffeine in the management of different neurodegenerative disorders, focusing on AD and PD. The current findings suggest that caffeine produces potent antioxidant, inflammatory, and anti-apoptotic effects against different models of neurodegenerative disease, including AD, PD, and other neurodegenerative disorders. Caffeine has shown strong antagonistic effects against the adenosine A2A receptor, which is a microglial receptor, and strong agonistic effects against nuclear-related factor-2 (Nrf-2), thereby regulating the cellular homeostasis at the brain by reducing oxidative stress, neuroinflammation, regulating the accumulation of α-synuclein in PD and tau hyperphosphorylation, amyloidogenesis, and synaptic deficits in AD, which are the cardinal features of these neurodegenerative diseases.

## 1. Introduction

Neurodegenerative diseases are characterized by their sudden onset and progression and are pathologically characterized by chronic loss of function and death of specific neuronal cells [1]. Morphologically, neuronal cell loss is associated with gliosis, and misfolding and accumulation of proteins, leading to abnormal extracellular and intracellular filamentous deposits in specific cell types, showing the main characteristics of neurodegenerative diseases [2]. One of the features responsible for neuronal cell loss is elevated oxidative stress, which is defined as an unevenness between the amounts of reactive oxygen species (ROS) produced and the extracellular and intracellular antioxidant defense systems, which are responsible for scavenging the ROS [3]. The antioxidative system passes through different phases during the neonatal stage, resulting in a relatively lower-grade cellular defense in preterm infants than term infants [4]. Some areas of the brain are prone to the effects of oxidative stress; these neuronal cells are called vulnerable cells, and the phenomenon is called neuronal vulnerability [5]. For example, neuronal cells in the frontal cortex, entorhinal cortex, amygdala, and hippocampus (CA1 region) are the most vulnerable to Alzheimer’s disease (AD)-associated degeneration. In contrast, in Parkinson’s disease (PD), the dopaminergic neurons in the substantia nigra are the most-affected neurons [6]. The different brain regions exhibit variable sensitivity to the elevated oxidative stress in different neurodegenerative disorders, reflecting the difference in the etiology of each disorder [7], although the cells involved in the pathogenesis of neurodegenerative diseases share a common sensitivity to elevated oxidative stress [8]. Neuroinflammation also plays a critical role in the execution of neuronal cell loss and neurodegenerative disorders [9]. Several factors may cause neuroinflammation: Oxidative stress, neurological trauma, redox iron, and accumulation of Aβ [10]. Subsequently, neuroinflammation may induce the secretion of different inflammatory cytokines, which activate τ hyperphosphorylation in the brain and are not phosphorylated under normal physiological conditions [11]. The well-known neurodegenerative disorders are AD and PD. AD is characterized by the aggregation of Aβ, τ hyperphosphorylation, neuroinflammation, and synaptic loss [12]. PD is also called a movement disorder characterized by dopaminergic neurodegeneration, accumulation of α-synuclein, and levy bodies [13]. Another life-threatening neurodegenerative disease is traumatic brain injury, which triggers neurodegeneration and AD-like pathological conditions [14]. For years, extensive research has focused on the exploration of compounds that may reduce elevated oxidative stress and its consequences for neurological diseases [15]. Among the different compounds, natural compounds have received considerable attention due to their safety, affordability, and availability [16]. Caffeine is a potent psychostimulant agent used around the world and in different settings in diverse models of neurodegenerative diseases [17]. Herein, we collected the recent studies conducted on the role of caffeine in the management of neurodegenerative diseases and the underlying mechanisms are presented. Special focus is placed on AD and PD, and particularly on the cellular antioxidant mechanisms executed by the nuclear-related factor-2 (Nrf-2) and its associated genes, and the microglial receptor adenosine A2A. Moreover, we have shed light on the effects of caffeine in the management of other toxins, such as cadmium-, aluminum-, and lipopolysaccharides (LPS)-induced neurodegenerative conditions.

## 2. Methodological Approaches

The review article provides a comprehensive review focused on the effects of caffeine against neurodegenerative diseases, mainly Alzheimer’s and Parkinson’s diseases. The motivation for the compilation was our previous studies on caffeine. Our studies have suggested that caffeine is a neuroprotective agent [18,19]. We searched for articles reporting the neuroprotective effects of caffeine in different settings, both in in-vivo and in-vitro models of neurodegeneration, focusing on AD and PD. The publications were collected from different independent databases using the keywords: “Neuroprotection”, “neuroprotective”, and “caffeine” for the search. For analyzing the literature, the abstracts were extensively studied and summarized. All types of articles based on experimental animals used for neurodegenerative diseases were included in the current review. Comparisons between the caffeine and control groups were included, where the control group was injected with a physiological saline/placebo. Administration of drugs, route of administration, duration of the dose, and the toxic compounds used to develop these models were not considered. All the studies covering the effects of caffeine in animal and cellular models of neurodegeneration focused on AD and PD were included, including 1-methyl-4-phenylpyridinium (MPP+), lipopolysaccharides (LPSs), and rotenone. Several types of cellular models were considered, including mouse hippocampal HT-22 cells and human neuroblastoma SH-SY5Y cells.

## 3. Sources of Caffeine, Administration, and Pharmacological Effects

Caffeine is the main constituent of coffee, tea, and soft drinks [20]. The usage of caffeine from beverages in Sweden, Britain, and Finland (which are the highest tea-consuming countries in the world) ranges from 100 and 400 mg per individual per 24 h (approximately) [21]. Caffeine is a known psychostimulant drug that is an important part of everyday life due to its effects on cognitive performance and alertness [22]. Most of the dietary caffeine is provided by beverages and foods (chocolate) [23]. Coffee contains a relatively higher amount of caffeine than other beverages, and the amount consumed per individual is dependent on age [24]. Usually, adults drink more coffee compared to the young, who prefer chocolate [24,25]. Caffeinated energy drinks and beverages are mostly used by young individuals and teenagers [26]. The use of sweetened carbonated beverages starts in childhood, reaches a peak in young adult life, and gradually drops with the progression of age [27]. Extensive studies have suggested the protective potentials of caffeine against several animal and cellular models of neurodegenerative diseases [28]. The most studied and known effects of caffeine are antioxidant effects [28], antagonistic effects against A2AR [29]. The antioxidant effects of caffeine are mainly based on its impact against endogenous antioxidant regulators, such as nuclear factor erythroid 2-related factor 2 (Nrf-2) and heme oxygenase-1 (HO-1), and by regulating the level of lipid peroxidation (LPO) and reactive oxygen species (ROS) [28]. Caffeine has been suggested to act as an antioxidant and ROS scavenger [30], preventing lipid peroxidation, reducing oxidative DNA damage [31], and showing immunomodulatory effects under oxidative stress in the mouse brain [28]. The anti-oxidant effects of caffeine were reported to be responsible for the anti-inflammatory and anti-neurodegenerative effects of caffeine, which are thought to be adenosine-receptor-dependent [32]. Caffeine has been administered intraperitoneally for different durations in different models, based on the duration of treatment, animal model, and toxin used for the study. Thus, no single dose has been used for the evaluation of neuroprotective potentials of caffeine.

## 4. Pathological Bases of Oxidative Stress and Neuroinflammation in AD and PD

**Figure 1:**
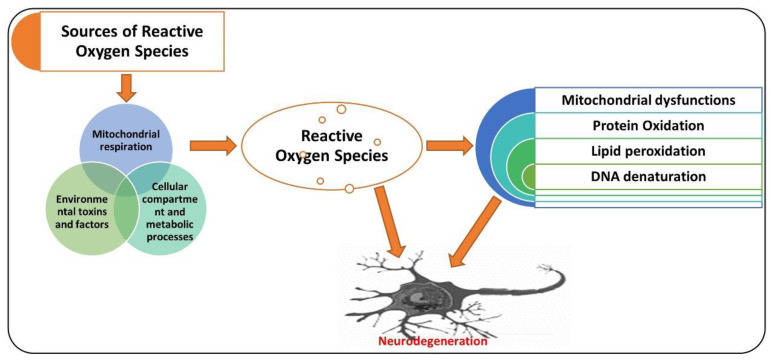
A simple illustration showing the role of reactive oxygen species (ROS) in neurodegenerative diseases. ROS may be produced from different sources, causing the mitochondrial dysfunction, oxidation of proteins, lipid peroxidation, and DNA denaturation, leading to neurodegeneration. The red arrow shows the processing. Red pointing arrow is showing the inducing effects.

Neurodegenerative diseases, as diverse group ailments, are characterized by progressive loss of neuronal cells in the targeted brain regions [33]. The pathophysiology of neurodegeneration is not yet fully understood; however, elevated oxidative stress has been indicated as one of the major factors responsible for the degeneration of neuronal cells [34,35]. Elevated oxidative stress may induce cellular damage, impair the DNA repair system, and trigger mitochondrial apoptosis, all of which are likened with aging and neurodegeneration [36,37]. Oxidative stress is caused by an imbalanced redox status, involving either production of a higher amount of ROS or dysfunction of the endogenous antioxidant mechanism responsible for the regulation of ROS [38]. The ROS is a group of molecules produced from oxygen that have a short life and are highly reactive because of their incomplete valence electrons [39]. Oxidative stress produced at the cellular level is called cellular oxidative stress, generated either by endogenous or exogenous systems [40]. Exogenous sources of ROS generation include ultraviolet radiation, drug metabolism, environmental toxins, certain chemicals, and the by-product of metabolic processes [41]. Endogenously, ROS are produced from the action of mitochondrial and non-mitochondrial enzymes, such as xanthine oxidase (XO), nicotinamide adenine dinucleotide phosphate (NADPH) oxidase (Nox), and cytochrome P450 [42]. Normally, 2% of the total cellular mitochondrial O_2_ consumption is associated with the generation of ROS, including O_2_ [43,44]. Oxidative stress is modulated by several factors, such as antioxidant gene nuclear factor-E2-related factor 2 (Nrf-2), a short-lived protein that functions as a transcription factor [45]. Nrf-2 regulates the level of several genes involved in xenobiotic metabolism, having antioxidant and anti-inflammatory effects [11]. Genes regulated by Nrf2 include heme oxygenase-1 (HO-1) and superoxide dismutase (*SOD1*), and enzymes playing a role in the glutathione degradation, such as glutathione S-transferase and glutathione cysteine ligase catalytic subunit (GCLC) [46,47]. Dimethyl fumarate (DMF), a specific inducer of Nrf-2, has shown promising neuroprotection effects in different models of neurodegeneration by upregulating the expression of Nrf-2 and its associated genes, such as HO-1 [48]. Apart from the reactive species, the reactive nitrogen species (RNS), including nitric oxide (NO), also contribute to neuronal cell death and neurodegeneration [49,50]. N-methyl-d-aspartate (NMDA)-type glutamate receptors have been associated with the reactive oxygen and nitrogen species produced in the central nervous system. These are activated in response to foreign invaders or the death of neuronal cells [51]. The activation of NMDA receptors causes increased influx of Ca^2+^ ions, which produce ROS and activate neuronal NO synthase (nNOS) [52]. The attachment of the NO group to the cysteine thiols of the targeted proteins results in the formation of S-nitrosoproteins (SNO-Ps), which may regulate the functions of protein [53]. The mitochondria produce free radicals, prominently ROS, in response to mitochondrial oxidative metabolism, xenobiotics, inflammatory cytokines, and bacterial pathogenesis. Also, in response to excessive calcium influx through abnormal malfunctioning of calcium regulating proteins and ion channels underlies the pathogenesis of AD, which has been discussed in details elsewhere [54]. Also, one species of ROS, superoxide anion, reacts with free radical NO to form the toxic compound peroxynitrite (ONOO–) [55]. The peroxynitrite oxidizes are a different sort of macromolecules, including lipids, DNA, and proteins. NO can directly nitrosylate macromolecules without involving any other molecule. It can also deactivate the respiratory enzymes and reduce the production of ATP, disturbing the energy supply and cellular homeostasis as a whole [56]. Apart from Nrf-2, there is a group of receptors known as adenosine receptors (ADO-Rs), which play a pivotal role against elevated oxidative stress and neuroinflammation [57]. Adenosine is a potent biological mediator produced form the active cells by diffusion or produced by the decomposition of ATP [58]. Adenosine modulates the activities of different cells, including neutrophils, neuronal cells, platelets, and smooth muscle cells [59]. Four adenosine receptors (ADO-Rs) (A1, A2A, A2B, and A3) have been uncovered to date [60]. Notably, A2A-R has been suggested as the main receptor rescuing the brain against neuronal trauma, ischemia, and hypoxic conditions [8]. It has been suggested that A2A-R defers apoptotic cell death in human neutrophils [61] and reduces serum-deprived apoptosis in PC12 cells [62]. Agents that may regulate the expressions of Nrf-2 and A2A-R in a biological system may reduce the elevated oxidative stress and neuroinflammation [63], although several natural and chemical compounds have shown promising therapeutic potential against different neurodegenerative diseases [64]. Here, we present the antioxidant effects of caffeine (Figure 1).

## 5. Pathophysiology of Alzheimer’s Disease (AD)

AD is a chronic neurodegenerative disease associated with oxidative stress [65], accumulation of hyperphosphorylated tau [66], and aggregation of amyloid plaques [67]. Several factors may aid in the progression of AD, such as age; genetic factors; pre-existing ailments, such as diabetes mellitus; cardiovascular disease; nutrition; and lifestyle, which are thought to play roles in its pathogenesis [68]. Besides the aggregation of amyloid-beta (Aβ1–42) plaques and hyperphosphorylation of tau, other factors contributing to the pathogenesis of AD include elevated oxidative stress, neuronal inflammation, and apoptotic cell death [69]. The pathogenesis of AD is not limited to the neuronal system; it also involves the microglial, astrocytic, and infiltrating cells from the peripheral nervous system, which aid in neurodegeneration [70]. So, the immune system plays a significant role in linking neurodegeneration with the neuroinflammatory process, executed by the activated microglial cells [71]. The neuroinflammation may lead to misfolding and hyperphosphorylation of tau and formation of amyloid-beta oligomers [72,73]. The formation of Aβ oligomers induces neurodegeneration, synaptic dysfunction, and memory dysfunction in AD patients [74]. Another main contributor to the pathogenesis of AD is elevated oxidative stress, as the AD patient presents a higher level of oxidative distress linked with the aggregation of Aβ and hyperphosphorylation of tau [75]. Extensive research studies have indicated the critical role of biometals including zinc, iron, and copper in neurodegenerative conditions and Alzheimer’s disease [76]. In agreement with those suggestions, there is a higher affinity binding for copper and zinc on the N-terminal domains of amyloid-beta and amyloid precursor protein (APP) [77]. In contrast, copper is a potential mediator of the highly reactive hydroxyl radical (HO^•^), subsequently adding to an elevation in the oxidative-stress-associated damage in the AD brain [78], according to the increased level of copper found in amyloid-beta plaques [79]. This elevated copper content is linked with the length of Aβ fragments, as Aβ (1–42) is relatively more toxic than Aβ (1–40) and is the most doubted candidate that produces hydrogen peroxide and reactive oxygen species [80]. A higher level of zinc has been associated with cognitive and memory-related regions of the brain [81], including the hippocampus, neocortex, and amygdala, which are the most affected regions in AD [6,82]. The attachment of zinc produces a highly organized conformational state of Aβ (1–40), leading to the production of the toxic, fibrillary, and aggregated form of Aβ [83]. Consequently, the inflammatory effect of non-soluble Aβ plaques disrupts zinc homeostasis, followed by the release of cerebral zinc, which is the main source of the of reactive oxygen species [84]. The release and accumulation of zinc induces zinc- and Aβ-mediated oxidative stress and neurodegeneration [85]. Phospholipids of the brain’s membranes are composed of polyunsaturated fatty acids, which are more exposed to the attack of free radicals [86]. This double binding permits removing hydrogen ions and triggers lipid peroxidation, which is the critical feature in the degeneration of the neuronal system in AD [87]. This oxidation of proteins is significantly higher in the case of AD, as the oxidation of the brain can badly affect the enzymatic processes, which are important for the physiological functions of the neuronal and glial cells [88]. This phenomenon is mainly focused on glutamine synthetase and creatine kinase, which are the most sensitive to oxidative damage and are markedly reduced in AD brains [89], producing a change in glutamate concentrations and triggering cellular toxicity, whereas oxidative damage of creatine kinase may reduce the energy homeostasis in the brain [90]. The accumulation of amyloid proteins leads to neurofibrillary formation and intricacy [91]. Neurofibrillary tangles are known by their accumulation and tau hyperphosphorylation into paired helical filamentous structures [92]. Hyperphosphorylation is associated with oxidation via the microtubule-associated protein kinase, and through the activation of nuclear factor-κB, it links oxidative damage to the phosphorylation of tau [93]. Elevated oxidative stress activates the advanced glycation end products (AGEs) as a result of post-translational modification of genes that are produced when an amino group of one protein reacts with monosaccharides of the other group (non-enzymatically) [94]. The oxidation of the brain regions may affect the DNA by creating strand breaks, DNA-protein crosslinking, and sister chromatid exchange [95]. So, elevated levels of reactive oxygen species produce devastating effects and can be a critical initiator of cellular structures’ destruction and neurodegeneration [96]. Figure 1 shows the sources of reactive oxygen species and their effects on the execution of neurodegenerative conditions.

One of the main contributors in the pathogenesis of AD is mitochondrial dysfunction, as the healthy and functional mitochondria not only supports neuronal activity by providing sufficient energy for mitochondrial functions of neurons but also protects neurons by minimizing mitochondrial oxidative damage [97]. Mitochondria are the major energy source providing ATP through oxidative phosphorylation to maintain the neuronal physiology and homeostasis [98]. Mitochondria play a role in the production of essential iron–sulfur center and heme in neurons and are implicated in the presynaptic transmitter biosynthesis in synapses. Mitochondria provide important buffering machinery to regulate calcium homeostasis during the signal transmission, which is of prime importance to excitable cells such as neurons. Neurons are the cells, having a long life span as the organism, and mitochondria acting as safeguards in protecting the neuronal cells under various stressing conditions [99]. So, it is not surprising that disturbances of mitochondrial functions are closely related to the pathophysiology of neurodegenerative diseases, including AD. The mitochondrial dysfunction may arise as a result of mitochondrial DNA abnormalities, mutation of nuclear proteins that interact with mitochondria. In most cases, it is not clear where the mitochondria sit concerning the disease progression that causes neuronal loss and death, and there are still arguments regarding the question of whether mitochondrial dysfunction is necessary for the execution of neurodegeneration in AD [100]. A complete discussion on the role of the mitochondrial dysfunction in the pathophysiology of AD has been presented elsewhere [101].

**Figure 2:**
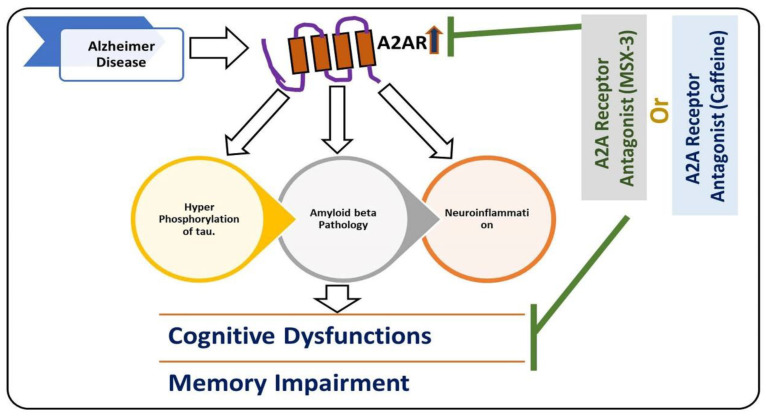
Role of A2A receptor in the execution of Alzheimer’s disease (AD) pathology. A diagram highlighting the role of microglial receptor A2A in the hyperphosphorylation of tau, accumulation of amyloid-beta, and neuroinflammation in AD pathology. The upregulation of the A2A receptor is responsible for AD pathology, and inhibition of the A2A receptor may reduce the hyperphosphorylation of tau, reduce amyloid-beta, and cause neuroinflammation. The reduction in hyperphosphorylation of tau and accumulation of amyloid-beta neuroinflammation rescue cognitive dysfunction and memory impairment. 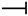, used for inhibition, the pointing arrow is used for induction.

Another main mediator of neuroinflammation is the transcription factor nuclear factor erythroid 2-like 2 (Nrf2) [102]. Nrf2 induces the expression of antioxidants and cytoprotective genes, which provoke an anti-inflammatory response that is critical for the healing process [103]. Apart from the different sorts of kinases (like MAP kinases), there are some receptors such as adenosine receptors that play a role in neuroinflammation, such as adenosine A2A receptors [104]. At the microglial level, the expression of A2AR is usually low; it activates with brain insults and facilitates the release of the inflammatory cytokines [105], and changes the microglia into amoeboid shapes [106]. Conversely, A2AR antagonists suppress microglia activation, as highlighted previously [104]. Figure 2 depicts the role of A2A in AD.

## 6. Antioxidant and Neuroprotective Effects of Caffeine against AD

Previous studies have presented different neuroprotective approaches using different models of AD, where different receptors and kinases have been targeted [107]. Agents relieving oxidative stress by boosting the endogenous antioxidant system and reducing lipid peroxidation have shown promising effects [108]. Among the different types of receptors, the microglial receptor A2A has been identified as playing a significant role in the activation of microglial cells and the release of inflammatory cytokines [109]. The selective adenosine A2A receptor antagonist (MSX-3) has shown promising effects in APPswe/PS1dE9 mice by reducing amyloid-beta accumulation [110]. In another study where the A2AR was deleted from the THY-Tau22 mice, the findings indicated that the deletion of A2ARs protects the mice from tau-pathology-induced deficits in terms of spatial memory and long-term hippocampal depression [111]. Collectively, the results suggest that adenosine A2A R plays a crucial role in AD pathology [112]. Recently, several studies have highlighted the role of caffeine (a non-specific antagonist of adenosine A2AR) in regulating reactive oxygen species, neuroinflammation, and other factors responsible for neuronal cell loss [28]. In PS1/APP transgenic mice, the administration of caffeine may reduce memory impairment by regulating the expression of brain-derived neurotrophic factor (BNDF) and tropomyosin receptor kinase B (TrkB) [113].

**Figure 3:**
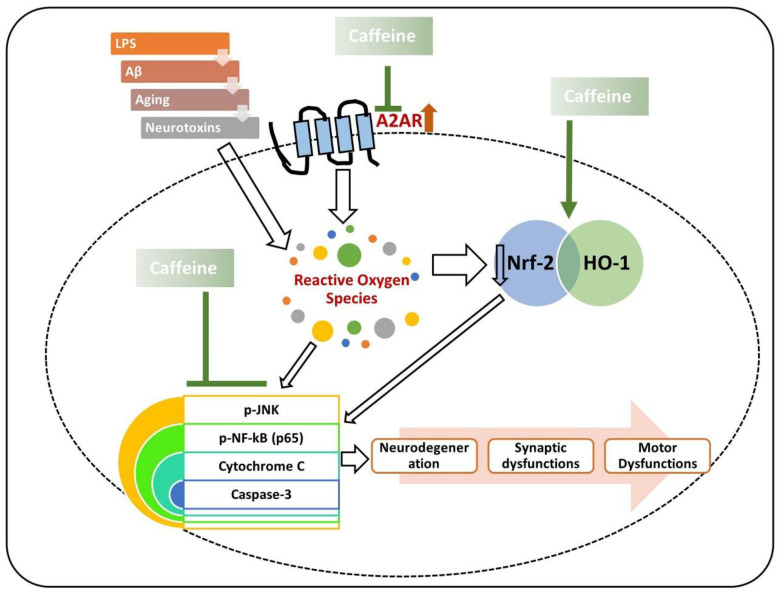
Role of caffeine in the management of neurodegeneration. The rescuing effects of caffeine against neurotoxins and age-related neurodegeneration. Caffeine suppresses the expression of the A2A receptor and upregulates the expression of nuclear factor erythroid 2-related factor 2 (Nrf-2), thereby regulating the inflammatory mediators (phospho-c-Jun n-terminal kinase (p-JNK), phospho-nuclear factor-kappa B (p-NF-kB)), apoptotic markers (cytochrome C, and caspase-3), synaptic dysfunctions, and neurodegeneration. Green arrows are used for the beneficial effects of caffeine. The arrows with white background are showing the inducing effects.

Similarly, in another study, chronic caffeine intake was found to potentially regulate the expression of BDNF glial fibrillary acidic protein (GFAP) in AD, thereby regulating AD pathology in the mouse brain [114]. Most recently, we reported that caffeine controls the AD-like pathological changes in mice by regulating the expression of Nrf-2 and TLR-4-induced glial-cells-mediated neuroinflammation and apoptotic cell death [115]. Similar results were obtained when D-galactose-treated mice were treated with caffeine; the findings showed that caffeine markedly reduced p-JNK-induced inflammation, synaptic, and memory dysfunction in mice [19]. From the collective findings, we conclude that caffeine may reverse the AD pathology by modulating neuroinflammation, oxidative stress, and apoptotic neuronal loss (Figure 3).

## 7. Effects of Caffeine against Other Neurotoxin Models of AD

Apart from the main models of AD, other models have been used for research related to AD, and the best known of which is the LPS-induced model [11]. Neurodegenerative diseases are disorders for which no cure has been developed to date [116]. Generally, the underlying factors involved in the progression and onset of disease are still poorly understood [117]. So, many efforts have been aimed at unveiling the pathophysiology of Alzheimer’s disease [118]. Neuroinflammation is a key mediator involved in the execution of neurodegeneration [9]. Several lines of studies have suggested that the death and injury of the neuronal cell may induce an inflammatory process, which leads to cell death [119]. It is crucially important to induce inflammatory conditions in models of neurodegeneration to analyze its underlying mechanisms and consequences [120]. Neuroinflammation may be influenced by different mechanisms, one of which is LPS [121]. LPS is present in the cell membrane of Gram-negative bacteria, targeting the toll-like receptor (TLR) 4 and acting on other related receptors [122]. The TLR4 activated by LPS induces a series of downstream effectors, such as myeloid differentiation primary response protein 88 (MyD88) and TRIF-related adaptor molecule (TRAM) [123]. The activation of these molecules can activate transcription factors and inflammatory cytokines [123]. Most of the studies related to neuroscience employed LPS to induce neuroinflammation by activating microglial cells, as neurons also have the TLR4 receptor [124]. The activation of TLR4 receptors leads to neuroinflammation and the release of inflammatory mediators [125]. So, the effects of caffeine against LPS-induced neurodegeneration have been extensively evaluated [126]. In a study, caffeine was injected using various doses in young rats with LPS over two to four weeks. The injection of LPS activated the microglial cells, but caffeine reduced the number of activated microglia within the hippocampus of LPS-treated rats [126]. We evaluated the effects of caffeine against LPS-induced inflammation and neurodegeneration [28]. Our findings suggested that caffeine may reduce elevated oxidative stress by regulating the levels of Nrf2 and HO-1 in caffeine co-treated mice. We found elevated expressions of TLR4, phospho-nuclear factor-kappa B (p-NF-kB), and phospho-c-Jun n-terminal kinase (p-JNK) in the LPS-injected mice brains; these were markedly reduced in the caffeine co-treated mice brains. We also found enhanced expressions of Bcl-2-associated X, apoptosis regulator (Bax), and caspase-3, and low expression of B-cell lymphoma 2 (Bcl-2) in the LPS-injected mice. Notably, these were significantly reversed in the caffeine co-treated mice. Similarly, the expressions of synaptic markers in the caffeine-co-treated mice were enhanced. The collective findings suggested that caffeine may reduce oxidative stress, neuroinflammation, and synaptic dysfunctions in the LPS- treated mice [115]. Caffeine has also shown neuroprotective effects against cadmium-induced oxidative stress, neuroinflammation, and cognitive dysfunction in mice. Cadmium (Cd), which is a non-biodegradable heavy metal, affects the major organs following either acute or chronic exposure [127]. To analyze the effects of caffeine against cadmium-induced neurodegeneration, we injected caffeine (30 mg/kg/per day intraperitoneally (i.p) against cadmium (5 mg/kg). Our results suggested that caffeine may reduce cadmium-induced oxidative stress, as revealed from the ROS and lipid peroxidation (LPO) assays and by regulating the level of Nrf-2 and HO-1, which act as endogenous antioxidant regulators. The expression of 8-dihydro-8-oxoguanine (8-OXO-G), which is produced as a result of DNA damage, is significantly enhanced in the case of neurodegenerative diseases [128]. According to our findings, the expression of 8-OXO-G was reduced in the caffeine-injected mice compared to the cadmium-treated group. Cadmium reduced the expression of activated microglial cells, as shown by the reduced expression of GFAP and Iba-1 in the experimental mice. Similarly, the expressions of the inflammatory and synaptic markers were significantly regulated in the caffeine-treated mice [129].

Aluminum (Al) is a known neurotoxin associated with various neurodegenerative diseases, such as AD and PD [130]. It is used in the preparation of different types of utensils and pharmaceuticals, and in the preparation of anti-gastric medication and antiperspirants, from where it gains entry into the human body [131]. Aluminum enters into the brain through high-affinity receptors causing neurological changes resulting in learning and cognitive dysfunctions in humans and experimental animals [132]. Due to its specific chemical structure and characteristics, it binds to the phosphate groups of RNA and DNA, changing the topology of DNA and influencing the expression of various genes that are critical for brain physiology [133]. In order to analyze the effects of caffeine against aluminum-induced neurodegeneration, caffeine, and nicotine were co-treated against aluminum-induced neurodegeneration. The behavioral results suggested that caffeine and nicotine co-administration had more pronounced protecting effects against aluminum-induced learning and memory impairment. The co-treatment of caffeine and nicotine also reduced aluminum-induced neurodegeneration in the hippocampus and the eosinophilic plaques in the striatum, whereas nicotine alone still showed mild gliosis in the striatum. Conclusively, they demonstrated that the co-treatment of caffeine and nicotine could reduce neurodegeneration and cognitive and memory dysfunction in the aluminum-injected model [130].

## 8. Pathophysiology of Parkinson’s Disease (PD)

**Figure 4:**
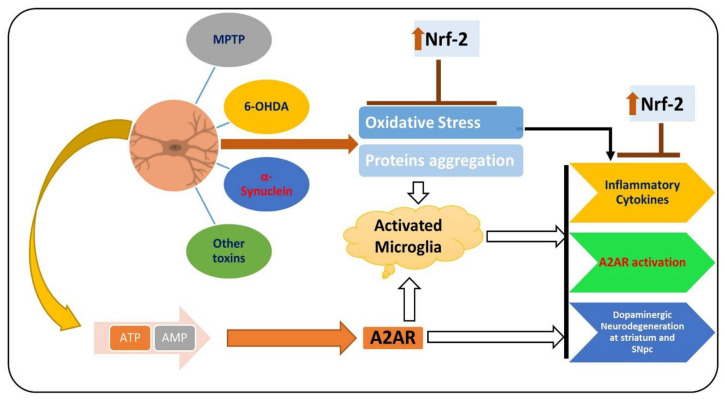
Role of A2A receptor and Nrf-2 in the pathophysiology of PD. MPTP, 6-OHDA, α-synuclein, and other PD-inducing toxins induce oxidative stress, consequently downregulating the expression of Nrf-2 and upregulating the expression of the A2A receptor. Elevated oxidative stress and the activation of the A2A receptor trigger neuroinflammation and dopaminergic neurodegeneration. 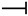, used for inhibition, the pointing arrow is used for the induction.

PD is a chronic and progressive neurodegenerative disease, manifested between the fifth and seventh decade of life, with resting tremor, muscle rigidity, bradykinesia, and abnormal postural reflexes [134]. PD affects 6 million people around the globe, which is expected to double in the coming 20 years [135]. The prominent pathological hallmarks of PD are loss of dopaminergic neurons in specific brain regions (substantia nigra and striatum) and the presence of Lewy bodies in the distinct neuronal compartments [136,137]. Extensive studies have focused on unveiling the pathological bases of the disease for almost two centuries since James Parkinson explored the disease in 1874 [138]. To date, there are no cure for PD, so effective treatments for this disease are urgently needed [139]. Several factors are involved in the pathophysiology of PD [140]. PD affects the neuronal system through several mechanisms, one of which is elevated oxidative stress, which notably affects the brain of PD patients by suppressing the endogenous antioxidant system and increasing the lipid peroxidation [64]. Apart from the elevated oxidative stress, activated microglial cells and the subsequent release of the inflammatory mediators have also been shown to play a prominent role in the execution of neuronal cell loss in PD [141]. The pharmacological inhibition of A2AR activates dopamine receptor-2, suppresses neuroinflammation by regulating the level of dopamine, and relieves the symptoms of PD [142]. The elevated oxidative stress and reduced level of dopamine due to the activation of A2AR in the substantia nigra and striatum of PD patients induce overall neuroinflammation and PD symptoms [143]. Elevated oxidative stress may enhance lipid peroxidation and suppress the endogenous antioxidant mechanisms responsible for scavenging the reactive oxygen species [144]. As mentioned previously, Nrf-2 is a transcription factor responsible for the regulation of multiple cellular processes [145]. Several studies have shown that in PD, the expression of Nrf-2 is suppressed, so the downstream such as HO-1 are affected, including elevated oxidative stress and neuroinflammation [146]. The elevated oxidative-stress-activated microglial cell and release of the inflammatory mediators trigger the loss of dopaminergic neurons, ultimately triggering PD symptoms [147]. Figure 4 shows the role of Nrf-2 and A2A receptor in PD.

## 9. Effects of Caffeine against PD-Related Neuroinflammation and Oxidative Stress

Although extensive research has focused on the development of candidate drugs for the treatment and management of PD, no effective drugs have yet been approved [64]. The only approved drug is dopamine replacement therapy, which relieves the symptoms, having a wide range of adverse effects in increased neurotoxicity and neuroinflammation, and deregulating thiols metabolism [148]. In recent years, many plants-derived flavonoids and other compounds have shown promising health benefits in PD patients, predominantly by regulating the elevated oxidative stress and neuroinflammation [149]. Among the different compounds used against PD animal models, caffeine has received considerable attention. A study conducted on the 6-OHDA -induced PD model showed that caffeine significantly regulated the expression of tyrosine hydroxylase (TH), TNF-α, IL-1β, and histone deacetylase (HDAC), and modulated the locomotor effects [150]. Caffeine markedly reduced the expression of gamma-aminobutyric acid (GABA) and enhanced the paw strength in MPTP-treated mice. Similarly, in the MPP+-treated SH-SY5Y cells, caffeine activated PI3K/Akt signaling and prevented apoptotic cell death by regulating the p-JNK and ERK signaling [151,152]. Caffeine also regulated PD-like pathology in α-synuclein-induced mice by modulating macroautophagy by improving the microtubule-associated protein LC-3, reducing the receptor protein sequestosome 1 (SQSTM1/p62) and chaperone-mediated autophagy (CMA), and by regulating the expression of LAMP2A [153]. To analyze whether the neuroprotective effects of caffeine are A2AR-dependent in MPTP-treated mice, the effects of caffeine were compared in wild-type (WT) and A2AR gene global knockout (A2A KO) mice, as well as in CNS cell type-specific (conditional) A2AR knockout (cKO) mice. In WT and heterozygous A2AR KO mice, caffeine treatment markedly regulated the level of dopamine in the striatum in the MPTP-treated mice.

Conversely, in A2AR global KO mice, caffeine had no significant effects on MPTP-induced neurotoxicity. In forebrain neuron A2AR cKO mice, caffeine lost its stimulating effects, whereas its neuroprotective effects remained in the case of astrocytic A2AR cKO mice; both the effects on locomotion and neuroprotective effects were undiminished. Collectively, the study suggested that the neuroprotective effects of caffeine in the MPTP-induced mouse model are dependent on the A2A receptor [154].

**Figure 5:**
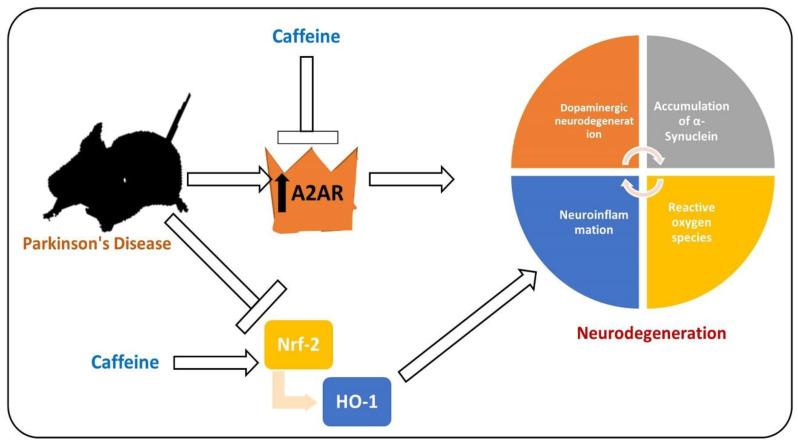
Effects of caffeine against Parkinson’s disease (PD)-like pathological changes in the mouse brain. Reduced expression of Nrf-2 and activated level of A2A receptor in a mouse model of PD. Caffeine shows regulatory effects against A2A and Nrf-2, thereby rescuing the mice against neurodegeneration and memory impairment. 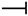, used for inhibition, the pointing arrow is used for the induction.

The neuroprotective potentials of caffeine have been studied in intrastriatal 6-OHDA-injected rats. The findings suggested that after two weeks of 6-OHDA administration, the rats exhibited specific rotational behavior due to morphine administration. The findings suggested an increased number of apomorphine-induced rotations in 6-OHDA-injected rats compared to the sham-operated rats. A significant effect was noted in 6-OHDA-lesioned rats when caffeine (10 and 20 mg/kg, i.p., daily for 14 days) was treated. The administration of 6-OHDA induced a loss of striatal neurons in the ipsilateral side (75–85%) compared to the contralateral side. There was a marked reduction in the levels of noradrenaline in the ipsilateral side of the 6-OHDA group (62%), which was not reversed in caffeine-treated rats. Similarly, caffeine significantly restored the level of dopamine in the ipsilateral side of 6-OHDA-injected rats, showing a significant recovery with the administration of caffeine. As a whole, the data suggested the protective potentials of caffeine in the rat model of PD, indicating the use of caffeine as an A2A receptor antagonist in the management of PD-associated pathological changes [155] (Figure 5).

## 10. Antioxidant and Neuroprotective Effects of Caffeine in In Vitro PD Models

Alongside in-vivo studies, some in-vitro studies have been conducted to strengthen the hypotheses related to the neuroprotective effects of caffeine. Researchers have evaluated the neuroprotective mechanisms of caffeine using human dopaminergic neuroblastoma SH-SY5Y cells for the induction of PD-like pathological changes in the cells. The neurotoxins used were 1-methyl-4-phenylpyridinium (MPP+, a bioactive form of MPTP), 6-OHDA, and rotenone. Collectively, they suggested that caffeine dose-dependently suppresses the apoptotic cell loss induced by serum/retinoic acid (RA) deprivation, in MPP+-, rotenone-, and 6-OHDA-treated SH-SY5Y cells. Caffeine reduced the caspase-3 activity induced by serum/RA deprivation and 6-OHDA administration; caffeine reduced apoptotic and fragmented nuclei.

Similarly, the phosphorylation of AKT was enhanced after 60 min of caffeine administration. The PI3K inhibitors (wortmannin and LY294002) inhibited the neuroprotective potentials of caffeine in the cellular model of PD, indicating that the effects conferred by caffeine are PI3K-dependent. The results suggested that MAPKs such as Erk1/2, p38, and JNK are not upregulated by caffeine, showing that MAP kinases are not involved in the protective mechanisms of caffeine. Collectively, the results show that the neuroprotective effects of caffeine are due to the regulation of the PI3K/Akt pathway. The findings reinforce the studies conducted on animal models, showing that caffeine may rescue the mouse brain against PD-related neurodegeneration [152].

## 11. Research Gap, Future Perspectives, and Considerations

Although caffeine has shown potent anti-inflammatory and antioxidant effects both in vitro and in vivo in animal studies, the evidence is inconclusive of its clinical outcomes in humans. Currently, evidence supporting the use of caffeine as drugs in subjects with neurodegenerative diseases is insufficient. There is an intensive need for human trials on caffeine to obtain more conclusive evidence. Although several studies have been conducted on the role of coffee (not caffeine) in managing different diseases in humans, one such study was conducted on multiple sclerosis. The effects of coffee consumption were observed on concentration while performing tasks, expanding attention span, and a better-structured daily routine [156]. Another study was conducted on the effects of coffee on neurocognitive performance (NCP) in HIV- and HCV-infected humans. The results indicated a positive correlation between elevated coffee intake (ECI) (three or more cups of coffee per day) and NCP in case of verbal fluency, psychomotor speed (coding), and decision-making functioning. They indicated that coffee intake might preserve the neurocognitive functioning in people living with HIV and HCV [157]. Other studies on the association between habitual coffee consumption and liver fibrosis [158], depression [159], hearing [160], and cognition indices [161] highlighted the beneficial effects of coffee. A study on the impact of caffeine intake on PD progression was conducted, which showed that healthy individuals with regular caffeine consumption had a lower risk of PD during follow-up observation. The outcomes included dyskinesia, motor performance, and symptom onset. Caffeine-consuming individuals showed a markedly reduced rate of PD symptoms [162]. More studies are underway to unveil the underlying mechanisms to introduce diet- or medicines-based drugs and advice.

Conversely, studies have shown that caffeine may cause migraine and affect the quality of sleep, mainly by activating the brain system [163]. The side effects and dosing frequency have not been fully established, so a clearer understanding and exploration are needed. Caffeine has shown promising neuroprotective effects in different models of neurodegenerative disease, covering AD and PD. Coffee or caffeine consumption was found to relieve the symptoms associated with or pathological consequences of neurodegenerative diseases. Caffeine has shown neuroprotection effects by reducing oxidative stress, neuroinflammation, and apoptotic cell death in different models of neurodegeneration [28]. The antioxidant effects of caffeine have been attributed to its impact on the expression of Nrf-2 and inhibition of microglial receptor A2A [29,164]. The transcription factor Nrf-2 and the microglial receptor A2A have been shown to play prominent roles in the pathophysiology of neurodegenerative diseases, and regulation of these factors has shown promising health benefits in neurodegenerative diseases. Interestingly, caffeine has shown dual effects against neurodegeneration by regulating the expressions of Nrf-2 and A2A receptor.

## 12. Conclusions

Previously, we conducted several studies on the role of caffeine in the modulation of neurodegenerative disease, where we used cadmium and LPS as inducers of neurodegeneration [18,28]. Our findings, together with those of other studies conducted on the role of caffeine in the management of neurodegenerative disorders, have indicated that caffeine produces strong antioxidant and neuroprotective effects against different diseases. The neuroprotective effects of caffeine are solely based on its pronounced effects against elevated oxidative stress and neuroinflammation. The antioxidant effects are achieved by its impact against the reduced expression of Nrf-2, as confirmed by our colleague, showing that silencing of Nrf-2 in the HT-22 cells may result in loss of the neuroprotective effects of caffeine, suggesting that the antioxidant effects of caffeine are Nrf-2-dependent [129]. The anti-neuroinflammatory effects of caffeine are achieved mainly by suppressing the activated microglial receptor adenosine A2A, as confirmed by studies. The A2AR was knocked out in mice, revealing that the anti-neuroinflammatory effects of caffeine are lost when the receptor A2A is deleted [154]. Collectively, the therapeutic approaches based on the pharmacological inhibition of the A2A receptor and regulation of oxidative stress may provide symptomatic relief against devastating neurodegenerative conditions such as AD and PD.

## Abbreviations

AD	Alzheimer’s Disease
PD	Parkinson’s Disease
MPTP	1-methyl-4-phenyl-1,2,3,6-tetrahydropyridine
MPP+	1-methyl-4-phenylpyridinium
Nrf-2	Nuclear factor erythroid 2-related factor 2
6-HO-1	Heme oxygenase-1
OHDA	6-hydroxydopamine
HDAC	Histone deacetylase
TH	Tyrosine hydroxylase
Aβ	Amyloid beta
LPS	Lipopolysaccharides
TrkB	Tropomyosin receptor kinase B
